# Assessment of Language and Literacy Teachers’ Distance Teaching in COVID-19 Lockdown Time

**DOI:** 10.3389/fpsyg.2021.762732

**Published:** 2021-11-30

**Authors:** Yi Huang, Jinjin Lu

**Affiliations:** ^1^Department of Psychology, Faculty of Social Studies, Masaryk University, Brno, Czechia; ^2^Institute for Research of Children, Youth and Family, Faculty of Social Studies, Masaryk University, Brno, Czechia; ^3^Department of Educational Studies at Academy of Future Education, Xi’an Jiaotong-Liverpool University, Suzhou, China

**Keywords:** COVID-19, language and literacy teachers, assessment, professional development (PD), distance teaching and learning

## Introduction

Due to the global pandemic of coronavirus disease 2019 (COVID-19), most governments temporarily suspended the traditional offline school education as one measure to stop the epidemic spreading, and more than 1.7 billion students have suffered from this (Gouëdard et al., 2020). Under such a context, Unesco advocated the distance education model (Miao et al., 2020). This dataset was built with the purpose of exploring and assessing the language and literacy teachers’ distance teaching (LLTDT). We developed and further assessed it for two reasons.

First, although there are some research tools that aim to examine the teaching behaviors and learning achievement in the period of COVID-19, very little was known from the perspectives of the teachers, particularly language and literacy teachers in mainland China. Language and literacy learning is essential for children to be successful in their future work and life (Chall et al., 1990). Chall et al. (1990) emphasized the importance of literacy-related environmental supports of school children (5–10 years old), such as those from the school teachers, parents, and communities, which would help them to enhance their language and literacy skills and benefit them in their life-long learning, rather than the direct improvement of their cognitive skills in a short-time. They argued that socioeconomic status of the students and their environmental backgrounds would significantly influence on their language and literacy learning. According to the theory of Chall et al. (1990), from the perspective of the teachers, the pedagogical expertise of lessons organization, supportive resources selection, and proper assessment for the students have significant influences on the language development and literacy learning of the children. When the parents and families are experiencing hardships, the language and literacy skills of the children would be poorer than those who are not. In this case, when the children and parents are involved in the unforeseen pandemic, language and literacy teaching is necessary and vital to investigate.

More importantly, compared with the traditional face-to-face teaching, experience and affordance of distance-teaching should be taken into more considerations. The development of literacy ability of the children, such as reading, writing, and communication, is essential for them to participate actively in their schools, homes, communities, and places of work (Luke and Freebody, 1997). For children at an early stage of their learning, the teachers have been regarded as the critical persons for their literacy development (Leu et al., 2013). Mantei and Kervin (2018) pointed out that pedagogical expertise of the teachers will influence children through designing and delivering literacy lessons, selecting supportive resources, and making judgments based on the assessment data.

The second reason for the importance of LLTDT assessment is, the teachers are believed to be in the front line for researching their practice as a model for their professional development. Professional Development: Support for Teaching and Learning (Department for Education and Employment, 2000) points that only the teachers know what they need to improve in professional development. However, when the COVID-19 pandemic sweeps the world, it brings the tremendous challenges, uncertainties, and hardships for the teachers, children, and parents. There are seen in a global educational context, particularly for those who are in the marginalized groups. Besides the financial crisis, many teachers have to experience the challenges of transmitting from a traditional face-to-face teaching model to deliver their teaching in a remote and blended way. In this pandemic period, the digital proficiency of the teachers, the skills of selecting online resources and their needs could influence teaching and learning motivation, efficiency, and outcomes of the students. For researchers, needs of teachers and their digital experience could provide first-hand data as an evidence base to drive for professional development programs in both the national and international educational contexts.

We combined the theoretical models used by the previous researchers (Chall et al., 1990; Botzakis and Malloy, 2006; Leu et al., 2013). Distance teaching is investigated in the three aspects: the preparedness of teachers of online teaching, learning achievement, and learning motivation for the perspectives of teachers. The first aspect is theorized by Chall et al. (1990) and Botzakis and Malloy (2006). Chall et al. (1990) viewed from a pedagogical perspective, which provides a backbone to assess the LLTDT from digital preparation for organizing lessons, supportive resources for digital distance teaching, and teacher-perceived learning outcomes of the students in distance teaching and learning environment. Botzakis and Malloy (2006), who used the international research correspondents (IRCs) to see teacher preparation in the different countries and regions, such as Australia, Canada, and Chile. The report indicated that the novice teachers would face more challenges in the implementation of standard teaching preparation. The other two aspects were theorized by Leu et al. (2013). A model of Leu et al. (2013) has been tested in the United States. Neither of the models are used by the teachers in mainland China in a situated context.

## Materials and Methods

### Design

The measurement was developed in collaboration with the teachers, research partners, and researchers for the second phase of the big international project, which aimed to examine the needs, challenges, and perceptions of teachers on the learning achievement of the students in the lockdown. The ethics was gained in February 2020 at the principal researcher’s institute. The research population was recruited from Wuhan, China, which has first reported the outbreak of COVID-19 in the West. First, the research assistant (RA) sent all the research information to the local primary schools to obtain the agreement of individual school managers and principals. Then, the RA sent the consent forms and an online survey link to the personal emails of the teachers. Due to strict lockdown in Spring academic semester in 2020, the participants only could access an online survey to complete. The consent forms were sent to the participants to complete successfully before starting the survey. That is, without signing the recent forms, the participants could not activate the online link of the survey. The data collection started in March 2020 and finished in December 2020. Before the closing date, the RA sent a reminder to the teachers who have not completed the survey.

The participants were selected based on the following criteria:

(1) both mentally and physically healthy;
(2) teaching in Wuhan City, China, amid the pandemic period; and
(3) experience distance teaching and blended teaching.

Socio-demographic information was collected (age, gender, teaching experience, and service training experience) to ensure the internal and external validity of the sample. The survey of teachers was original in English, and we used a back translation method to ensure its high linguistical standard so that the teachers could have a good understanding of the contend (Brislin, 1970). The 25 scaled items were rated on a Five Likert Scale (1932), from “Strongly Disagree” to “Strongly Agree” (1 = Strongly Disagree to 5 = Strongly Agree) to express to what extent they disagreed or agreed. The details of the Teachers’ Survey are in Supplementary Material.

### Materials

As mentioned, our questionnaire to investigate LLTDT focused on three aspects: (1) digital preparation for organizing lessons, (2) supportive resources for digital distance teaching, and (3) assessing the learning outcomes of students in distance teaching and learning environment.

For examining the digital preparation of the teachers for lessons organization, three domains were considered: teachers’ planning and preparation, technology use, and distance teaching challenges. The example items of related domains were “I was well prepared to move to remote learning when learning from home began,” “I had the skills to use the technologies and software available to develop and deliver classwork to the learners from the beginning of the “learning from home” period,” and “It has been difficult to track the literacy progress of all my students during this time.” In addition, we required the responses of teachers toward the perceived developmental support to investigate the supportive resources for distance teaching, for instance, “I have been provided with sufficient/useful professional learning and support to carry out my language and literacy teaching during these times.” Finally, regarding the assessment of teachers of the outcomes of students, teacher-perceived learning achievement (e.g., “the students improved teamwork capacity”), and motivation were viewed (e.g., “counting the online video watching time into grade credits motivated students”).

### Participants

In this study, 250 Chinese teachers participated in this research. Of the total sample, 29.2% (*N* = 73) participants were men and 70.8% (*N* = 177) were women. Most of the teachers (*N* = 93, 37.2%) were at the age from 30 to 39 years old. Only 4% (*N* = 10) of teachers were from 50 to 59 years old. The largest portion of teaching experience was 6–10-year (*N* = 72, 28.8%). All the participants adopted distance teaching during the survey time.

For each item, the participants responded from 1 to 5. The average score for each item ranged from 3.23 to 4.00. Except for the median scores of the item “students improved teamwork capacity” and the item “students had not gained too much” were three, the median score for other items were four. The details of socio-demographic information and the test results are presented in Table 1.

**TABLE 1:** Social-demographic information of teachers and the descriptive statistics for the measurement.

	**N (%)**	**Mean**	**Median**	**Standard deviation**
Male	73 (29.2%)			
Female	177 (70.8%)			
**Age**				
From 20 to 29 years old	86 (34.4%)			
From 30 to 39 years old	93 (37.2%)			
From 40 to 49 years old	61 (24.4%)			
From 50 to 59 years old	10 (4.0%)			
**Teaching experience**				
1–5 years	63 (25.2%)			
6–10 years	72 (28.8%)			
11–20 years	56 (22.4%)			
21–30 years	49 (19.6%)			
31–40 years	10 (4.0%)			
**Grade**				
First grade of primary school	81 (32.4%)			
Second grade of primary school	83 (33.2%)			
Third grade of primary school	86 (34.4%)			
**Measurement results**				
Item1	3.72	3.72	4	0.936
Item2	3.89	3.89	4	0.802
Item3	3.86	3.86	4	0.839
Item4	3.74	3.74	4	0.919
Item5	3.98	3.98	4	0.828
Item6	3.62	3.62	4	0.955
Item7	3.62	3.62	4	0.911
Item8	3.75	3.75	4	0.912
Item9	3.62	3.62	4	0.862
Item10	3.23	3.23	3	1.09
Item11	3.76	3.76	4	0.917
Item12	3.72	3.72	4	0.86
Item13	3.9	3.9	4	0.805
Item14	3.11	3.11	3	1.16
Item15	3.62	3.62	4	1
Item16	3.87	3.87	4	0.841
Item17	3.86	3.86	4	0.766
Item18	3.94	3.94	4	0.829
Item19	3.89	3.89	4	0.896
Item20	3.98	3.98	4	0.811
Item21	3.96	3.96	4	0.865
Item22	3.93	3.93	4	0.842
Item23	4	4	4	0.867
Item24	3.87	3.87	4	0.845
Item25	3.8	3.8	4	0.841

### Power Analysis

Previous experience recommended the sample size of 5–10 respondents per item (Tinsley and Tinsley, 1987; Shin and Kim, 2007). Therefore, the sample size of 250 participants was adequate for our 25-item measurement. Moreover, according to the power analysis for the two-tailed correlative relationship, type I error rate at 1%, type II error rate at 10%, expected correlative effect size at 0.30, and at least 158 participants should be included (Cohen et al., 2014). In conclusion, the sample size in this research had sufficient statistical power.

## Data Analyses and Results

### Validity Test

#### Factor Analysis

This study planned to adopt exploratory factor analysis (EFA) to confirm the dimensions of the measurement. Before running EFA, it was necessary to examine the suitability of the data. The Kaiser–Meyer–Oklin measure of sampling adequacy was 0.931, which was over the recommended value of 0.60. In addition, Bartlett’s test of sphericity result was significant (*p* < 0.001), which also suggested the adequacy of data sampling for factor analysis (Pallant, 2011).

Initially, the EFA results suggested a four-factor model explaining 50.1% variance. According to the scree plot, a three-factor solution was more applicable. The three-factor structure (as shown in Table 2) explained 47% variance. Except for the three items, “I feel confident that I designed meaningful and appropriate remote language and literacy learning experiences for my learners” (item 24), “Our school needed to distribute equipment and resources to students before they could attempt remote learning” (item 4), and “I had the skills to use the technologies and software available to me to develop and deliver classwork to my learners from the beginning of the “learning from home” period” (item 6), all the other item-factor loadings were excessive to the criterion of 0.45 (Tabachnick et al., 2007). The correlation between factor 1 and factor 2, that between factor 1 and factor 3, and that between factor 2 and factor 3 were 0.678, 0.535, and 0.568, respectively.

**TABLE 2:** Item-factor loadings of language and literacy teachers’ distance teaching (LLTDT) measurement.

	**Factor**			
**Item**	**1**	**2**	**3**	**Uniqueness**
Item12	0.821			0.434
Item13	0.756			0.442
Item11	0.69			0.481
Item8	0.689			0.496
Item7	0.634			0.51
Item9	0.599			0.596
Item5	0.55			0.563
Item25	0.53			0.504
Item3	0.529			0.555
Item17	0.508			0.596
Item10	0.498			0.753
Item2	0.478			0.612
Item16	0.452			0.559
Item24	0.400			0.475
Item4	0.349			0.614
Item22		0.833		0.375
Item23		0.738		0.43
Item21		0.682		0.511
Item20		0.601		0.434
Item18		0.565		0.474
Item19		0.482		0.525
Item15			0.681	0.468
Item1			0.600	0.394
Item14			0.455	0.832
Item6			0.443	0.626

*“Minimum residual” extraction method was used in combination with an “oblimin” rotation.*

### Reliability Test

#### Internal Consistency

According to the general criteria for internal consistency (Ursachi et al., 2015), the total score of the measurement suggested that the internal consistency was high (Cronbach’s alpha = 0.932). Besides, we also tested the internal consistency for each sub-factor. Except for the internal consistency of factor 3 (Cronbach’s alpha = 0.668) was only at the acceptable level, the coefficients of Cronbach’s alpha values of factor 1 (alpha = 0.911) and factor 2 (alpha = 0.870) indicated high internal consistency reliabilities.

#### Item Analysis

We examined the homogeneity of each item in LLTDT measurement by the approach of item-rest correlation, which referred to the computation of the correlative effect size between the interested item and the rest of the scale. Generally, the correlative coefficient should be above 0.30 (Ferketich, 1991). The results suggested the item “students had not gained too much” (item 14) was problematic due to the low item-rest correlation. Besides, we analyzed the contribution of each item for internal consistency of a scale by an item-drop approach. As expected, the internal consistency described by Cronbach’s alpha value would decrease if we dropped an item with a contribution. Again, the same item showed a negative effect on the internal consistency of measurement. And the other items contributed to the internal consistency.

## Data Usage and Applications

The dataset is useful because we establish a measurement to assess the LLTDT during the special “learning from home” period. COVID-19 pandemic has caused the impossibility to continue the traditional offline teaching activities. Both the teachers and students have faced the new challenge of distance education. Under the framework of the theories of Chall et al. (1990); Botzakis and Malloy (2006), and Leu et al. (2013), we developed an instrument to investigate LLTDT under the Chinese context. This measurement helps us to understand the LLTST and teachers needed in distance teaching more deeply:

(1) LLTDT can be assessed by three dimensions: teaching expertise, teacher-perceived learning motivation, and teacher-student connectedness. From the perspective of internal consistency, the first two dimensions are relatively stable;
(2) teaching expertise can be measured by the questions about distance teaching planning and preparation, the assessment of learning achievement of the students, and perceived developmental support for teaching expertise;
(3) teacher-perceived learning motivation related questions belong to an independent cluster;
(4) teacher-student connectedness can be measured by the questions about tracking of the teachers in the learning process of students, contact frequency and the subjective overall feelings of teachers of gaining of the students. However, it is worth noticing that this dimension may be unstable crossing different samples based on the internal consistency computation results. As it is important for the teachers to track the learning process of students, we suggest further research to design more items to probe teach-student contact in the distance education context;
(5) the measurement structure based on the factor analysis suggests the significance of language and literacy planning and preparation of teachers for distance education and developmental support (Mantei and Kervin, 2018), as well as the assessment of teachers for the motivation and achievement of students. The results echo with previous research (Chall et al., 1990; Botzakis and Malloy, 2006; Leu et al., 2013); and
(6) the dataset provides sectional information for further correlative models, for instance, the construction of structural equation models to probe the mechanism to improve online learning achievement of the students or education efficiency of the teachers (Tsai et al., 2012; Sá and Serpa, 2020).

## Limitations

The measurement was developed based on a regional research. Thus, it is necessary to expand the sample to test the validity and measurement invariance crossing regions. In addition, there was an imbalance in gender of the participants. We suggest further research to expand the scale of male participants. Moreover, this research raises the consideration of teacher-student contact in distance teaching. It would be valuable to design a special instrument to assess the teacher-student digital communications.

## Data Availability Statement

The original contributions presented in the study are included in the article/Supplementary Material, further inquiries can be directed to the corresponding author/s.

## Ethics Statement

The studies involving human participants were reviewed and approved by the Masaryk University. The participants provided their written informed consent to participate in this study. Written informed consent was obtained from the individual(s) for the publication of any potentially identifiable images or data included in this manuscript.

## Author Contributions

YH and JL contributed to the data report. YH completed the development of the research tool and finished the report writing. JL provided the data analysis and provided supervision on report writing. Both authors contributed to the article and approved the submitted version.

## Conflict of Interest

The authors declare that the research was conducted in the absence of any commercial or financial relationships that could be construed as a potential conflict of interest.

## Publisher’s Note

All claims expressed in this article are solely those of the authors and do not necessarily represent those of their affiliated organizations, or those of the publisher, the editors and the reviewers. Any product that may be evaluated in this article, or claim that may be made by its manufacturer, is not guaranteed or endorsed by the publisher.
