# Supplementary material for: Assessment of Language and Literacy Teachers’ Distance Teaching in COVID-19 Lockdown Time
Source: Front Psychol. 2021 Nov 30;12:762732. doi: 10.3389/fpsyg.2021.762732 (PMC8669427; doi:10.3389/fpsyg.2021.762732)

**Questionnaire Section 1. Backgrounds**

Gender

1. male 2. female

Age

1. 20-29 years old

2. 30-39 years old

3. 40-49 years old

4. 50-59 years old

What is your teaching experience?

1. Less than 5 years

2. 6-10 years

3. 11-20 years

4. 21-30 years

5. 31-40 years

What is the grade you are teaching

1. Grade 1

2. Grade 2

Please describe your school

1. Independent

2. Government

3. Others

What is your geographical context?

1. City

2. Large regional town

3. Rural

4. Remote

Where are you working now

1. At school

2. At home

3. At both school and home

**Questionnaire Section 2. Language and Literacy Teachers’ Distance Teaching (LLTDT) Measurement**

*Note: A1 refers to "Aspect 1: Digital preparation for organizing lessons"; A2 refers to "Aspect 2: Supportive resources for digital distance teaching"; A3 refers to " Aspect 3: Assessment of students' learning outcomes in distance teaching and learning environment"*

|  | 1. Strongly  disagree | 2. Disagree | 3. Neither  agree nor  disagree | 4.  Agree | 5. Strongly agree |
| --- | --- | --- | --- | --- | --- |
| Item 1 (A1-planning/preparation).  I was well prepared to move to remote learning when learning from home began |  |  |  |  |  |
| Item 2 (A1-planning/preparation).  My confidence grew during my experience with remote learning |  |  |  |  |  |
| Item 3 (A1-planning/preparation).  I am better equipped to develop and deliver remote learning now |  |  |  |  |  |
| Item 4 (A1-planning/preparation).  Our school needed to distribute equipment and resources to students before they could attempt remote learning. |  |  |  |  |  |
| Item 5 (A1-planning/preparation).  In my school we have worked together/helped/learnt  from each other as we learned to deliver online literacy programs |  |  |  |  |  |
| Item 6 (A1-technology use). I had the skills to use the technologies and software available to me to develop and  deliver classwork to my learners from the beginning of the" learning from home" period |  |  |  |  |  |
| Item 7 (A3-learning achievement).  Students' mastery of knowledge was better than my expectation |  |  |  |  |  |
| Item 8 (A3-learning achievement)  Students' self-learning capacity improved |  |  |  |  |  |
| Item 9 (A3-learning achievement)  Students' communication skills improved |  |  |  |  |  |
| Item 10 (A3-learning achievement)  Students improve teamwork capacity |  |  |  |  |  |
| Item 11 (A3-learning achievement)  Students gained more practical skills |  |  |  |  |  |
| Item 12 (A3-learning achievement)  Students' learning interests improved |  |  |  |  |  |
| Item 13 (A3-learning achievement)  Students broaden their vision and knowledge |  |  |  |  |  |
| Item 14 (A3-learning achievement)  Students had not gained too much |  |  |  |  |  |
| Item 15 (A1-distance teaching challenges) It has been difficult to track the literacy progress of all my students during this time |  |  |  |  |  |
| Item 16 (A1-distance teaching challenges).  I have been able to follow the usual literacy program I developed for my class |  |  |  |  |  |
| Item 17 (A1-distance teaching challenges).  Being able to work from home allowed me to balance my changing workload |  |  |  |  |  |
| Item 18 (A3-learning motivation)  What do you think of attendance should be counted into credits? |  |  |  |  |  |
| Item 19 (A3-learning motivation)  What do you think of online teaching watching should be counted into credits? |  |  |  |  |  |
| Item 20 (A3-learning motivation)  What do you think of classroom performance should be counted into credits? |  |  |  |  |  |
| Item 21 (A3-learning motivation)  What do you think of presentations, classroom discussions and peer feedback are not counted into credits? |  |  |  |  |  |
| Item 22 (A3-learning motivation)  What do you think of joining in forum discussions, Q and A sessions and sharing resources online are not counted into credits? |  |  |  |  |  |
| Item 23 (A3-learning motivation)  What do you think of joining in forum discussions, Q and A sessions and sharing resources online are not given extra marks in the examinations? |  |  |  |  |  |
| Item 24 (A2). I feel confident that I designed meaningful and appropriate remote language and literacy learning experiences for my learners. |  |  |  |  |  |
| Item 25 (A2). I have been provided with sufficient/useful professional learning and support to carry out my language and literacy teaching during these times |  |  |  |  |  |

**Correlation Heatmap to describe the item-item correlation**


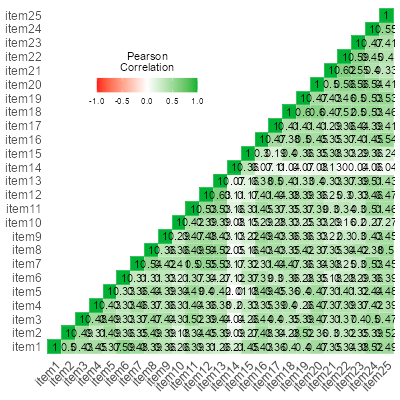

Supplement: Supplementary file 1 [file Data_Sheet_1.docx]
